# Association Between Metabolic and Atherogenic Indices and Circadian Blood Pressure Patterns in White-Coat Hypertension

**DOI:** 10.3390/medicina62020379

**Published:** 2026-02-14

**Authors:** Arzu Akgül, Cigdem Ikhlef, Çağatay Tunca, Mehmet Deniz Aylı

**Affiliations:** 1 Division of Nephrology, Department of Internal Medicine, Ankara Etlik City Hospital, 06710 Ankara, Turkey; cigdemmengus@hotmail.com (C.I.); d_ayli@hotmail.com (M.D.A.); 2 Department of Cardiology, Ankara Etlik City Hospital, 06710 Ankara, Turkey; md.tunca@gmail.com

**Keywords:** hypertension, atherogenic index, Triglyceride-Glucose index, uric acid, inflammatory markers

## Abstract

*Background and Objectives*: The risk of cardiovascular events rises when hypertensive patients fail to achieve sufficient blood pressure reduction during nighttime hours. This study examined the association between metabolic and inflammatory biomarkers and non-dipper patterns in patients with white-coat hypertension. *Materials and Methods*: A total of two hundred and forty-four (244) patients with newly diagnosed white-coat hypertension were included in the study. The study used ambulatory blood pressure monitoring to classify patients as dippers (n = 86) and non-dippers (n = 158). The study evaluated metabolic markers through triglyceride–glucose (TyG) index, atherogenic index of plasma (AIP) and uric acid measurements against inflammatory markers including neutrophil/lymphocyte ratio, platelet/lymphocyte ratio and monocyte/lymphocyte ratio. *Results*: The non-dipper group showed higher levels of uric acid (6.42 mg/dL vs. 5.65 mg/dL; *p* < 0.001), TyG index (8.82 vs. 8.51; *p* < 0.001), AIP (0.49 vs. 0.37 **; ** *p* < 0.001) and body mass index (26.0 kg/m^2^ vs. 24.1 kg/m^2^; *p* < 0.001) when compared to the dipper group. The inflammatory markers showed no significant variation between the groups. Uric acid showed the highest discriminative ability (AUC = 0.722). The research showed that each one mg/dL increase in uric acid levels was associated with 89% higher odds of non-dipper status (OR = 1.892; *p* = 0.002). A one-unit increase in the TyG index was associated with approximately 2.5-fold-higher odds. *Conclusions*: Levels of TyG index, uric acid, BMI and AIP were higher in patients with the non-dipper pattern compared to the dipper patients. The TyG index, uric acid levels, and BMI were independently associated with the non-dipper pattern in white-coat hypertension patients. These findings suggest that metabolic biomarkers may help identify individuals at higher risk for circadian blood pressure dysregulation.

## 1. Introduction

Research indicates hypertension stands as a major worldwide cause of cardiovascular disease because it affects 1.3 billion people [1]. The non-dipper pattern of blood pressure rhythm during the nighttime shows direct links to higher cardiovascular disease risks and death rates [2]. Hermida et al. demonstrated that cardiovascular risk is increased not only in hypertensive patients but also in normotensive subjects with a non-dipping pattern [3]. The non-dipper pattern affects approximately half of hypertensive patients who experience dangerous complications including target organ damage, heart attacks and strokes [4].

The cardiovascular risk factors, together with clinical presentation and treatment results between male and female patients, demonstrate significant variations [5]. Women face an underappreciated risk of heart disease because medical professionals commonly believe women have natural protection against cardiovascular disease, which results in different symptoms and inadequate medical care [6]. Although cardiovascular disease develops 7-to-10-years later in women compared to men, it remains the leading cause of death in women over the age of 65 [6]. Furthermore, emerging health challenges such as long COVID may impose additional constraints on obesity prevention in women, particularly during the postmenopausal period, potentially accelerating cardiometabolic risk trajectories and necessitating earlier intervention strategies [7]. The development of cardiovascular risk which occurs between adolescence and young adulthood requires healthcare providers to detect metabolic biomarkers at their earliest appearance [8]. People who live differently and choose to be adventurous will develop cardiovascular health problems during their early adulthood years [9].

In recent years, the importance of metabolic biomarkers has gained significant attention. Among these, the Atherogenic Index of Plasma (AIP) and the triglyceride-glucose (TyG) index have been frequently studied for cardiovascular risk assessment and their possible association with blood pressure patterns [1]. The TyG index serves as a surrogate marker for insulin resistance, while elevated uric acid levels reflect both metabolic dysfunction and potential endothelial impairment [10,11]. Ambulatory Blood Pressure Monitoring (ABPM) provides much more detailed information compared with office-based measurements and is therefore considered the gold standard for evaluating nocturnal blood pressure patterns [9]. Early identification of metabolic abnormalities may help detect individuals at risk of circadian blood pressure dysregulation before the development of sustained hypertension [11].

The pathophysiology of non-dipping blood pressure patterns involves multiple interrelated mechanisms, including alterations in autonomic nervous system activity, baroreflex sensitivity, endothelial cell function, sodium sensitivity, aortic stiffness, and renin–angiotensin–aldosterone system (RAAS) activity [10]. Notably, endothelium-dependent vasodilation has been shown to be impaired in non-dippers compared to dippers in hypertensive patients, suggesting that endothelial nitric oxide synthase (eNOS) dysfunction plays a key role in circadian blood pressure dysregulation [10]. Insulin resistance, a hallmark of metabolic syndrome, contributes to endothelial dysfunction by reducing nitric oxide bioavailability and promoting oxidative stress, which may further disrupt the decline in normal nocturnal blood pressure [11].

The current literature shows limited evidence about simple and reliable biomarkers which could help detect the non-dipper pattern in its early stages. Most existing studies have been conducted on small and heterogeneous populations [1]. The research needs to investigate which metabolic and inflammatory markers affect non-dipper pattern development in patients with white-coat hypertension who do not receive treatment. The identification of circadian rhythm disturbance mechanisms together with practical predictors will help scientists create new time-based therapeutic approaches [10,11,12]. The current research lacks sufficient data about sex-specific cut-off values and the ability to apply these findings to various population demographics.

Our research followed a hypothesis which stated that metabolic biomarkers show better predictive value than inflammatory markers for non-dipper pattern identification in patients with white-coat hypertension. The study evaluated the diagnostic accuracy of metabolic indicators such as TyG index, AIP and uric acid against the inflammatory ratios NLR, PLR and MLR for non-dipper pattern prediction. The study established sex-specific cut-off values which could serve as clinical reference points.

## 2. Materials and Methods

### 2.1. Study Design and Ethical Approval

This retrospective cross-sectional study analyzed pre-existing clinical data from the cardiology outpatient clinic records. All patient data were anonymized prior to analysis, and unique study identifiers were used to maintain confidentiality. Only authorized personnel had access to the deidentified dataset. The research used existing clinical data which was anonymized so the ethics committee granted permission to conduct the study without needing patient consent for this retrospective analysis. The use of patient data was approved by the hospital’s institutional review board and management team.

### 2.2. Study Population

Data were retrospectively extracted from the medical records of patients who underwent ABPM between January and June 2024 in the cardiology outpatient clinic. A total of 244 patients with white-coat hypertension were included. White-coat hypertension was defined as office blood pressure ≥ 140/90 mmHg with normal out-of-office blood pressure values according to the 2018 ESC/ESH guidelines and 2024 ESC guidelines [13,14]. Based on ABPMs, the normotension threshold was defined as <130/80 mmHg for the 24 h period, <135/85 mmHg for the daytime average, and <120/70 mmHg for the nighttime average [13,14].

The study included participants who met these criteria: age range from 18 to 65 years, patients with blood pressure readings ** ≥ **140/90 mmHg measured in the clinic but with 24 h ABPM average <130/80 mmHg, and patients with no history of antihypertensive medication use.

The study excluded participants with secondary hypertension, chronic kidney disease, heart failure, diabetes mellitus, chronic inflammatory disorders, active infection, malignancy, pregnancy and insufficient 24 h ABPM recordings.

### 2.3. Classification of Dipping Status

The circadian blood pressure pattern was determined by the percentage of nocturnal systolic blood pressure decline relative to the daytime mean [13,14]. Patients were classified as “dippers” if their nocturnal systolic BP fell by ≥10% compared to the daytime average [13,14]. A nocturnal decline of <10% in systolic BP was defined as a “non-dipper” pattern [13,14]. This classification was based solely on systolic BP according to the 2018 ESC/ESH guidelines; therefore, diastolic BP patterns were reported separately for descriptive purposes but did not influence group assignment. These diagnostic criteria and threshold values are consistent with the standardized recommendations for out-of-office blood pressure monitoring provided by the European Society of Cardiology [13,14].

### 2.4. Data Collection

The researchers documented all demographic information, and medical background and physical assessment results, through established standardized documentation tools. The calculation of Body Mass Index (BMI) required weight measurement in kilograms divided by height measurement in meters squared. The antecubital vein served as the blood collection site for venous blood samples which researchers obtained between 08:00 and 10:00 a.m. after patients fasted for at least 12 h.

### 2.5. Laboratory Measurements

The automated analyzer performed measurements of hemoglobin, neutrophils, lymphocytes, monocytes and platelets. The enzymatic colorimetric methods analyzed fasting glucose, creatinine and uric acid in blood samples. The lipid profile assessment used a homogeneous enzymatic colorimetric assay to determine total cholesterol, HDL, LDL and triglycerides. The bromocresol green method was used for albumin level determination.

### 2.6. Calculation of Metabolic and Inflammatory Indices

The calculation of TyG index required the following formula: ln [(fasting triglyceride (mg/dL) × fasting glucose (mg/dL))/2] [10]. The AIP was computed as log (triglyceride/HDL-cholesterol) [2]. NLR, PLR, and MLR were derived from absolute counts.

### 2.7. Ambulatory Blood Pressure Monitoring

Twenty-four-hour ABPM was performed using validated oscillometric devices (Schiller BR-102 plus, Schiller AG, Baar, Switzerland). Measurements were taken from the non-dominant arm with appropriately sized cuffs [2]. The devices were programmed to record every 15 min during the day (07:00–23:00) and every 30 min at night (23:00–07:00). The research used fixed time windows to detect day and night periods instead of using diary sleep and wake patterns, which researchers validate as an acceptable method when individual sleep data cannot be obtained [13].

Data were accepted as reliable if at least 70% of the readings were successful. Nocturnal decline (%) was calculated as [(mean daytime BP − mean nighttime BP)/mean daytime BP × 100] [1]. Day/night ratios were separately obtained for systolic and diastolic pressures. Morning surge was defined as the difference between the mean morning (07:00–10:00) and nighttime BP values. Differences between morning (07:00–10:00) and evening (19:00–22:00) periods were also analyzed. Pulse pressure (SBP–DBP difference) and threshold exceedances (SBP ≥ 140 mmHg, DBP ≥ 90 mmHg) were recorded.

### 2.8. Statistical Analysis

Statistical analyses were performed using SPSS version 28.0 (IBM Corp., Armonk, NY, USA) and MedCalc version 20.0 (MedCalc Software Ltd., Ostend, Belgium). Data distribution was tested with the Shapiro–Wilk test. Normally distributed variables were expressed as mean ± standard deviation (SD), while non-normally distributed data were presented as median (Q1–Q3). Between-group comparisons were conducted using Student’s *t*-test for normally distributed variables and the Mann–Whitney U test for non-normal variables. The analysis of paired comparisons between morning and evening data points used paired *t*-tests or Wilcoxon signed-rank tests. The analysis of matched categorical data employed McNemar’s test while independent categorical data analysis used the chi-square test. Correlations between biomarkers were assessed using Pearson’s correlation coefficient. Receiver Operating Characteristic (ROC) analysis was used to determine the diagnostic performance of each index, and optimal cut-off points were calculated using Youden’s index. AUC differences were evaluated with the DeLong test. The clinical utility of adding TyG index and uric acid to the base model was not evaluated using Net Reclassification Index (NRI) and Integrated Discrimination Index (IDI) due to the exploratory and retrospective nature of the study.

### 2.9. Logistic Regression Analysis

Independent predictors of the non-dipper pattern were identified using univariate and multivariate logistic regression. Candidate variables entered into the stepwise procedure included: age, sex, BMI, smoking status, TyG index, AIP, NLR, PLR, MLR, and uric acid. Variables with *p* < 0.20 in univariate analysis were entered into the multivariate model using a stepwise approach (entry criterion *p* < 0.05, removal criterion *p* > 0.10). Given the high correlation between TyG and AIP (r = 0.909), these indices were initially included together in the multivariate model; subsequently, due to non-significance, AIP was removed through the stepwise procedure, and VIF values were calculated for the final model. Four models were constructed:

- Model 1: Demographic factors
- Model 2: Demographic + metabolic indices
- Model 3: Additionally including inflammatory indices
- Model 4: All significant variables combined

Model fit was assessed using the Hosmer–Lemeshow test, and explanatory power with Nagelkerke R^2^. Multicollinearity was checked using variance inflation factor (VIF) values. Final model VIF values were: age = 1.12, sex = 1.08, BMI = 1.24, TyG = 1.31, uric acid = 1.19 (all <3, confirming the absence of multicollinearity).

### 2.10. Subgroup and Correlation Analyses

The researchers conducted subgroup evaluations according to sex distribution, which included 177 female participants and 67 male participants. Separate ROC analyses were performed for each group. The study investigated the relationship between nocturnal dipping percentage and biomarkers. Bonferroni correction was applied for multiple comparisons. The analysis of inter-biomarker relationships included multicollinearity assessment.

Statistical significance was set at *p* < 0.05. The *p*-values received two decimal places when greater than 0.01 but three decimal places when less than 0.01 and “*p* < 0.001” when appropriate.

## 3. Results

### 3.1. General Characteristics of the Study Population

A total of 244 patients with white-coat hypertension were included in the study. The mean age was 32.2 years. Most participants were female (n = 177, 72.5%), and about one-third were current smokers. Based on ABPM results, 35.2% of patients exhibited a dipper pattern (n = 86), while 64.8% had a non-dipper pattern (n = 158). There were no significant differences between groups regarding age or sex distribution. However, body mass index (BMI) was significantly higher in the non-dipper group (Table 1).

The lipid profile demonstrated substantial metabolic variations between these two groups. Non-dippers presented elevated levels of total cholesterol, LDL and triglycerides but their HDL levels remained lower. The triglyceride levels between dippers and non-dippers showed the most significant variation with dippers having 87.5 mg/dL and non-dippers having 120.5 mg/dL. Fasting glucose levels were similar between groups, though slightly higher in non-dippers. Uric acid levels were clearly elevated in non-dippers (Table 1).

**Figure 1:**
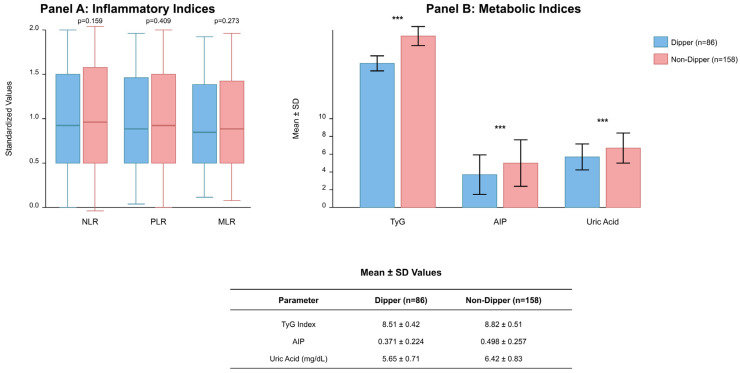
Comparison of inflammatory and metabolic biomarkers between dipper (n = 86) and non-dipper (n = 158) groups in patients with white-coat hypertension (N = 244). (**A**) Inflammatory indices: box plots displaying NLR, PLR, and MLR values with median (horizontal line), interquartile range (box), and whiskers extending to minimum and maximum values; no statistically significant differences were observed between groups (all *p* > 0.05). (**B**) Metabolic indices: bar graphs representing TyG index, AIP, and uric acid levels with error bars indicating standard deviation; all comparisons demonstrated statistically significant differences (*** *p* < 0.001 for each parameter). The table below presents mean ± SD values for metabolic parameters. Abbreviations: NLR, neutrophil-to-lymphocyte ratio; PLR, platelet-to-lymphocyte ratio; MLR, monocyte-to-lymphocyte ratio; TyG, triglyceride–glucose index; AIP, atherogenic index of plasma; SD, standard deviation.

NLR, PLR, and MLR values did not differ significantly between groups (all *p* > 0.05). The metabolic indices produced distinct results between the two groups. The TyG index measured 8.82 ± 0.51 in non-dippers while dippers had an average of 8.51 ± 0.42 which produced a highly significant result (*p* < 0.001). The AIP values measured higher in patients whose blood pressure did not dip (0.498 ± 0.257 vs. 0.371 ± 0.224, *p* < 0.001). The non-dipper group showed elevated uric acid levels at 6.42 ± 0.83 mg/dL compared to the dippers at 5.65 ± 0.71 mg/dL (*p* < 0.001). The three parameters demonstrated an excellent ability to detect non-dipper status through their combined results (Figure 1).

### 3.2. Independent Predictors of the Non-Dipper Pattern

The univariate logistic regression analysis demonstrated that BMI, TyG, AIP and uric acid levels showed significant relationships with occurrence of the non-dipper pattern. Given the high correlation between TyG and AIP (r = 0.909), AIP was excluded from the final multivariate model due to multicollinearity concerns. The multivariate models showed the following results:

- The risk of the non-dipper pattern increased by 15.8% for every 1-unit BMI increase.
- The risk of the non-dipper pattern increased by 2.5 times with each unit increase in TyG.
- The risk of the non-dipper pattern increased by 89.2% for every 1 mg/dL rise in uric acid levels.

Age and sex were not independent predictors. The final model showed a proper fit according to the Hosmer–Lemeshow test because the *p*-value exceeded 0.05 and the VIF values for all variables remained below 3, which indicated no multicollinearity problems (Table 2).

### 3.3. Diagnostic Performance

**Figure 2:**
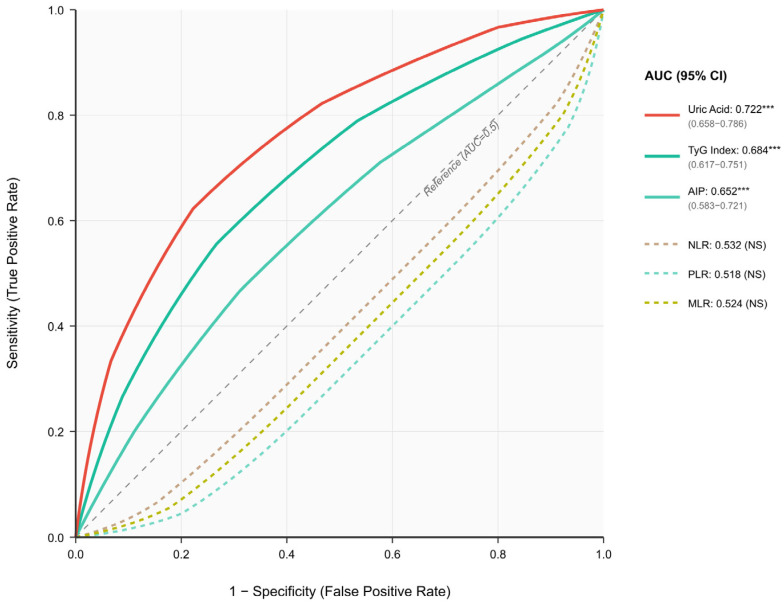
Receiver operating characteristic (ROC) curves for discrimination of non-dipper pattern (n = 158) from dipper pattern (n = 86) in patients with white-coat hypertension (N = 244). ROC curves display the diagnostic performance of metabolic indices (solid lines) and inflammatory markers (dashed lines) for predicting non-dipper status. Among metabolic indices, uric acid demonstrated the highest discriminative ability (AUC = 0.722, 95% CI: 0.658–0.786), followed by TyG index (AUC = 0.684, 95% CI: 0.617–0.751) and AIP (AUC = 0.652, 95% CI: 0.583–0.721); all three were statistically significant (*** *p* < 0.001). Inflammatory markers (NLR, PLR, MLR) showed no significant discriminative value with AUC values approximating 0.5 (all *p* > 0.05). Pairwise AUC comparisons using the DeLong test revealed that uric acid had significantly higher diagnostic accuracy compared with TyG index and AIP (*p* < 0.05 for both comparisons), whereas no significant difference was observed between TyG index and AIP. The diagonal dashed line represents the reference line (AUC = 0.5, no discrimination). Abbreviations: ROC, receiver operating characteristic; AUC, area under the curve; CI, confidence interval; TyG, triglyceride–glucose index; AIP, atherogenic index of plasma; NLR, neutrophil-to-lymphocyte ratio; PLR, platelet-to-lymphocyte ratio; MLR, monocyte-to-lymphocyte ratio; NS, not significant. *** *p* < 0.001. Pairwise *p*-values adjusted for multiple comparisons using Bonferroni correction.

ROC analyses showed uric acid produced the best diagnostic results with an AUC value of 0.722 (95% CI: 0.658–0.786). TyG (AUC = 0.684 **, 95% CI: 0.617–0.751 **) and AIP (AUC = 0.652 **, 95% CI: 0.583–0.721 **) showed moderate performance. None of the inflammatory indices were significant, as their AUC values remained near 0.50. The DeLong test revealed that uric acid achieved better diagnostic performance than TyG and AIP because it produced statistically significant results in both comparisons (*p* < 0.05). The study found that a uric acid level exceeding 5.95 mg/dL successfully identified non-dipper patients with 72.2% sensitivity and 68.6% specificity (Figure 2).

### 3.4. Sex-Specific Subgroup Analyses

The distribution of dipper and non-dipper patterns appeared equally common between male and female subjects (35% vs. 65% in both sexes). The best uric acid threshold for diagnosis proved to be 5.85 mg/dL in female patients and 6.15 mg/dL in male patients. The research established different cut-off points for TyG and AIP based on sex. The diagnostic power of uric acid exceeded TyG and AIP in both male and female patients. The DeLong test confirmed uric acid provided better diagnostic results than other biomarkers in both male and female patients (Table 3).

### 3.5. Correlations Among Biomarkers

**Figure 3:**
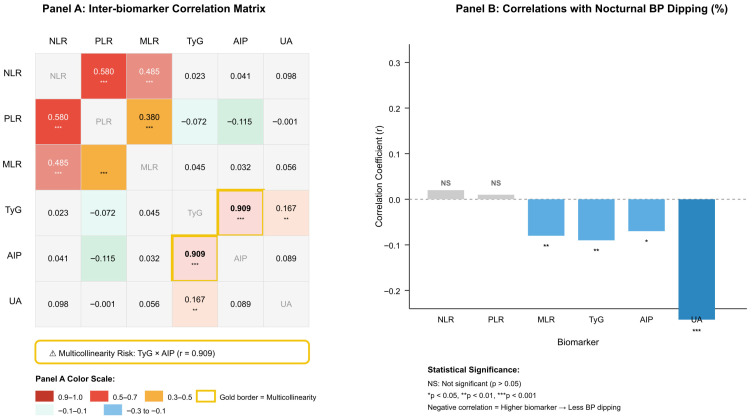
Biomarker intercorrelations and associations with nocturnal blood pressure dipping in patients with white-coat hypertension (N = 244). (**A**) Inter-biomarker correlation matrix displaying Pearson correlation coefficients among inflammatory markers (NLR, PLR, MLR) and metabolic indices (TyG, AIP, UA). Inflammatory markers showed moderate-to-strong intercorrelations (r = 0.38–0.58, all *p* < 0.001), whereas correlations between inflammatory and metabolic biomarkers were negligible (r < 0.12). The critical multicollinearity between TyG index and AIP (r = 0.909, *p* < 0.001) is highlighted with a gold border, indicating these variables could not be included simultaneously in multivariate regression models. (**B**) Bar graph showing correlation coefficients between each biomarker and the percentage of nocturnal systolic blood pressure decline. Negative correlations indicate that higher biomarker values are associated with reduced nocturnal BP dipping (i.e., non-dipper pattern). Uric acid demonstrated the strongest inverse correlation with nocturnal BP dipping (r = −0.264, *p* < 0.001), followed by TyG index (r = −0.09, *p* < 0.01), MLR (r = −0.08, *p* < 0.01), and AIP (r = −0.07, *p* < 0.05). NLR and PLR showed no significant associations with dipping status (*p* > 0.05). **Abbreviations:** NLR, neutrophil-to-lymphocyte ratio; PLR, platelet-to-lymphocyte ratio; MLR, monocyte-to-lymphocyte ratio; TyG, triglyceride–glucose index; AIP, atherogenic index of plasma; UA, uric acid; BP, blood pressure; NS, not significant. * *p* < 0.05, ** *p* < 0.01, *** *p* < 0.001.

The highest correlation with nocturnal dipping percentage occurred with uric acid measurements (r = −0.264, *p* < 0.001). The relationship between uric acid levels and the non-dipper pattern became stronger as uric acid levels rose, because it resulted in reduced blood pressure decreases during the nighttime. TyG and AIP showed similar negative correlations with nocturnal BP dipping (r = −0.09 and r = −0.07, respectively; both *p* < 0.05), but MLR produced a weak negative correlation. The study found no significant relationship between NLR and PLR values and dipping status (both *p* > 0.05). The analysis showed a strong positive correlation between TyG and AIP values (r = 0.909, *p* < 0.001) which indicated multicollinearity; therefore, these variables were not included simultaneously in the regression models (Table 3, Figure 3).

### 3.6. Morning–Evening Blood Pressure Variations

The evaluation of all participants as a group resulted in systolic pressure reducing by 5.9 mmHg and diastolic pressure reducing by 7.1 mmHg from morning to evening. The decline was more pronounced among dippers (systolic 11.3 mmHg; diastolic 12.1 mmHg). In non-dippers, reductions were only 2.8 and 4.6 mmHg, respectively. The average decrease in mean arterial pressure during the nighttime reached 12.8% in dippers but only 3.6% in non-dippers. The proportion of participants with morning systolic BP above 140 mmHg was 3.3%, which decreased to 0.8% in the evening. Similarly, the proportion with diastolic BP above 90 mmHg significantly declined in the evening (Table 4).

## 4. Discussion

This study investigated the effect of metabolic and inflammatory biomarkers on the non-dipper pattern in individuals diagnosed with white-coat hypertension. The relatively young mean age (32.2 years) and elevated prevalence of the non-dipper pattern (64.8%) in this cohort warrant clarification. Our study population comprised untreated white-coat hypertension patients undergoing initial ABPM assessment in an outpatient screening setting. This referral pattern—identifying individuals with newly recognized office hypertension who have not yet initiated treatment—naturally results in a younger, relatively healthy population with relatively higher non-dipper prevalence compared to published cohorts of treated hypertensive patients. Serum uric acid levels proved to be the most effective biomarker for detecting non-dipper patterns in patients. The TyG index and AIP showed predictive values for metabolic parameters but their strong relationship with each other requires careful interpretation in clinical practice. The study results show that inflammatory markers failed to provide any diagnostic value because circadian rhythm disturbances seem to stem from long-term metabolic changes instead of short-term inflammatory responses. Dippers exhibited a substantially greater morning blood pressure surge and their blood pressure readings differed substantially between day and night, highlighting the importance of circadian blood pressure patterns for cardiovascular risk assessment.

Our findings indicate that metabolic indices help clinicians predict non-dipper status. The non-dipper group showed higher TyG index values which corresponded to a 2.5-times-increased risk for each unit increase in the index, thus supporting the theory that insulin resistance disrupts normal blood pressure patterns throughout the day. Similarly, Sun and Liu recently reported TyG as a strong independent predictor in patients with non-alcoholic fatty liver disease (NAFLD) [15]. Our finding of higher AIP values in non-dippers also mirrors the results of Karayiğit et al., who identified AIP as an independent predictor in a large cohort of 1030 patients [2].

A notable finding was the strong relationship between TyG and AIP (r = 0.909), which created multicollinearity that eliminated AIP’s statistical significance in multivariate analysis. Sun and Liu encountered an identical problem between TyG and TyG/HDL-c values in their research [15]. The research by Asil et al. demonstrated that AIP levels were higher in dippers but diabetes comorbidities might affect this relationship [16]. The clinical utility of TyG as a screening tool for non-dippers surpasses AIP because TyG maintains its statistical significance and demonstrates better performance in AUC values. The evaluation of TyG index alone provides both methodological advantages and operational efficiency because both markers measure identical metabolic processes.

Our findings regarding non-dipper patients align with previous studies demonstrating elevated uric acid levels and strong predictive power. The research by Durak on 310 hypertensive patients demonstrated that patients with high uric acid levels displayed fewer dipper patterns and more reverse-dipper patterns [17]. Zhu et al. discovered that elevated serum uric acid levels predisposing individuals to the development of hypertension from a prehypertensive state through their research, which suggested that oxidative stress activation and endothelial dysfunction play a role in this relationship [18].

The sex-specific associations between serum uric acid levels and blood pressure patterns observed in our study are consistent with recent large-scale analyses [19]. Notably, uric acid levels have been found to play an important role as a biological mediator in the relationship between sex and cardiovascular mortality, with higher baseline levels in men compared to women [19]. These sex-based differences in cardiovascular mortality and the prognostic value of metabolic indices such as uric acid are critical factors that should be considered in clinical risk stratification [19].

Our findings regarding sex-specific differences in uric acid and metabolic indices align with the growing body of evidence suggesting that cardiovascular risk profiles vary significantly between men and women [5]. Medical research indicates that clinicians fail to detect heart disease risks in women because they have limited awareness about hormone protection in women, and because women present heart disease symptoms that differ from male symptoms [6]. Research studies which analyzed large datasets have demonstrated that death rates from all causes and heart disease show distinct patterns between men and women, thus medical practice needs sex-specific mortality thresholds [8,19].

Notably, conflicting findings exist regarding uric acid and non-dipper patterns in treated populations. Chotruangnapa et al. discovered that treated hypertensive patients with non-dipper profiles showed decreased uric acid levels [20]. These discordant results likely reflect differences in treatment regimens and patient characteristics rather than biological inconsistency. This study included participants diagnosed with white-coat hypertension and therefore not receiving any antihypertensive treatment, thus eliminating the possibility of drug effects on metabolic processes.

Our findings support the association between BMI and circadian rhythm patterns observed in previous research. The study results confirmed that obesity affects circadian rhythm patterns because BMI values were higher in non-dipper patients and BMI remained an independent risk factor. The study by Huart et al. established obesity together with salt sensitivity and metabolic problems as essential factors that create the non-dipper condition [21]. The study conducted by Chotruangnapa did not find any BMI variations between dipper and non-dipper participants because of possible differences in ethnicity and participant selection methods [20].

The multivariate model shows that uric acid and BMI operate independently to predict non-dipper status through different pathways where uric acid causes endothelial damage and activates the renin–angiotensin system, and BMI leads to insulin resistance and elevated sympathetic nervous system activity.

The more pronounced morning blood pressure surge observed among dippers in our study aligns with previous reports on physiological blood pressure responses. The study by Chu et al. demonstrated that untreated hypertensive patients who were dippers showed a greater morning systolic blood pressure elevation at 26.9 ± 12.6 mmHg compared to non-dippers at 15.9 ± 10.7 mmHg [22]. Although our values were lower (11.3 ± 4.2 vs. 2.8 ± 3.1 mmHg), the direction of difference was consistent. This difference is likely due to the inclusion of participants with normal ABPM results in our study.

The study by Renna et al. demonstrated the predictive value of morning surge in 1339 patients because it found that increases in systolic blood pressure above 35 mmHg during morning hours doubled the risk of cardiovascular events [23]. The 11.3 mmHg systolic blood pressure increase in our dipper-group patients indicated they had not yet entered a dangerous risk category.

Our night–day ratios were also in agreement with the literature. The systolic and diastolic ratios in non-dipper patients reached 0.98 and 0.94, which confirmed insufficient nocturnal blood pressure reduction. The research by Havelkova et al. shows that these ratios strongly link to cardiovascular risks and organ damage but warns that one-day ABPM tests could incorrectly identify 40% of cases [24]. The study results are affected by this monitoring limitation because we conducted our research using a single day of monitoring.

Inflammatory markers demonstrated no diagnostic utility for non-dipper prediction. The NLR, PLR and MLR values showed no group differences and their AUC values approached 0.50, which indicates that acute inflammation does not significantly affect non-dipper physiology. The research evidence supports the theory that chronic metabolic and neurohumoral mechanisms drive circadian rhythm disorders more than short-term inflammatory processes.

The uric acid thresholds we discovered for male and female patients matched the results from previous research. The study by Perticone et al. analyzed 1650 untreated hypertensive patients to establish optimal uric acid ranges of 4.8–5.2 mg/dL for women and 5.3–5.6 mg/dL for men to predict cardiovascular events [25]. The research supports the requirement for elevated uric acid thresholds in male patients since our study used thresholds above 5.85 mg/dL for women and 6.15 mg/dL for men.

The research by Liu et al. analyzed data from 27,075 participants to reveal how uric acid levels differ between male and female patients. Liu et al. demonstrated that uric acid levels maintained their independent link to hypertension in male patients but not in female patients [26]. The results from our study show uric acid performs well as a diagnostic tool for non-dipper status in both male and female patients, even if the participants were normotensive.

These findings suggest that the non-dipper pattern in white-coat hypertension patients may be a reflection of broader metabolic and endothelial dysfunction that differs between men and women [5,8]. Cardiovascular risk factors are known to be distributed differently between sexes in acute presentations, such as hypertensive crises and long-term clinical outcomes [8]. The development of cardiometabolic risk profiles, including blood pressure regulation and insulin resistance, during the transition from adolescence to young adulthood supports the notion that vascular health follows a sex-specific trajectory from early stages [8].

The similar TyG thresholds observed between men and women (>8.64 and >8.58, respectively) indicate that metabolic effects on circadian rhythm may be sex-independent. The equal percentage of dipper and non-dipper patients between male and female participants (35% vs. 65%) indicates that circadian rhythm problems affect both sexes equally.

The higher blood pressure readings in the morning compared to the evening demonstrate why healthcare providers should focus on morning blood pressure measurements. The research by Kuwabara et al. showed that elevated uric acid levels correspond to increased morning systolic blood pressure readings [27]. The results indicate that using morning blood pressure measurements together with uric acid thresholds that differ between sexes would enhance the ability to detect non-dipper patients.

### Limitations

This study has certain limitations. Firstly, the assessment of the circadian blood pressure pattern is based solely on a single 24 h ambulatory blood pressure measurement (ABPM). This technical limitation, commonly encountered in clinical practice, indicates that the dipping classification may change with repeated measurements, as emphasized in the literature [13]. Previous studies have reported that approximately 30–40% of patients may be reclassified when ABPM is repeated.

The retrospective design of the study prevented the evaluation of potentially important variables such as insulin levels, HOMA-IR, visceral fat ratio, muscle mass, dietary sodium intake, and sleep quality. Visceral adiposity is strongly associated with insulin resistance, systemic inflammation, and autonomic nervous system dysfunction, all of which may contribute to non-dipper blood pressure patterns. Similarly, reduced muscle mass (sarcopenia) has been linked to metabolic syndrome and cardiovascular risk, independent of total body weight. The lack of body composition analysis (such as bioelectrical impedance analysis or dual-energy X-ray absorptiometry) in this retrospective study precluded the assessment of these potentially important confounders. Consequently, the observed associations between metabolic indices and non-dipper status may be partially mediated or confounded by unmeasured differences in body composition between groups. Furthermore, the absence of waist circumference measurements precluded the calculation of certain biometric indices, such as the Visceral Adiposity Index (VAI). Similarly, biometric inflammation indices that incorporate anthropometric measurements—such as the Lipid Accumulation Product (LAP) and Cardiometabolic Index (CMI)—could not be calculated due to this limitation. These indices have been associated with subclinical inflammation and cardiovascular risk in previous studies, and their inclusion could have provided additional insights into the relationship between adiposity-related inflammation and circadian blood pressure patterns. Future prospective studies with comprehensive anthropometric assessments should evaluate the potential utility of these biometric inflammation indices in predicting non-dipper status. Although individuals with chronic kidney disease were excluded from the study and mean creatinine levels remained within normal limits, the lack of eGFR calculation represents a methodological shortcoming.

Although gender-specific threshold values for uric acid were determined in this study, the underlying mechanisms for these differences—such as hormonal effects (e.g., menopausal status) or lifestyle factors—could not be examined in detail [6]. The findings should be interpreted in light of the fact that women are often underrepresented or underestimated in cardiovascular risk assessments, which may limit the generalizability of the results [6].

Although it is known that the relationship between uric acid and cardiovascular mortality varies by gender, the retrospective nature of the study does not allow for a clear determination of the long-term prognostic value of these biomarkers [19]. Furthermore, the failure to assess external factors that could directly influence metabolic parameters, such as dietary habits, physical activity levels, and alcohol consumption, is another limitation in interpreting gender-specific differences [19]. Due to the exploratory and retrospective nature of the study, the additional contribution of the TyG index and uric acid to traditional risk factors could not be analyzed using the Net Reclassification Index (NRI) or Integrated Discrimination Index (IDI); prospective studies are needed in this regard.

The definition of daytime and nighttime periods with fixed time intervals may have affected the dipping classification, particularly in individuals with atypical sleep patterns. The lack of detailed lifestyle data (particularly sodium intake and physical activity) limits the assessment of the relationship between metabolic markers and circadian blood pressure patterns. Furthermore, the cross-sectional design of the study does not allow for the establishment of causal relationships.

Finally, the fact that the majority of the sample consisted of women (72.5%) and the relatively small size of the male subgroup (n = 67) may limit the generalizability of gender-specific findings. Furthermore, the limitation of the study population to the 18–65 age range excluded the older age group, which carries a higher cardiovascular risk. The reliability of the dipping classification could have been improved if multiple ABPMs had been possible.

Nevertheless, the study has some strengths. The examination of untreated white-coat hypertension patients excluded the effect of drugs on metabolic processes. Furthermore, the combined evaluation of multiple biomarkers and the definition of gender-specific threshold values increased the clinical value of the study. Prospective studies with repeated ABPMs in different populations are important for confirming these findings.

## 5. Conclusions

Focusing on the circadian rhythm rather than merely the measured blood pressure values appears to be a more significant factor in determining cardiovascular risk. Individuals diagnosed with white-coat hypertension are often excluded from lifestyle modifications and treatment evaluations. Consequently, the cardiovascular risk in these individuals, who exhibit a non-dipper pattern, is overlooked.

In our study the TyG index, BMI and uric acid levels independently predicted non-dipper patterns in participants. The diagnostic accuracy of uric acid proved highest among the three factors. The clinical use of uric acid would improve through the creation of separate thresholds for male and female patients. The results indicate that screening should concentrate on metabolic indicators because inflammatory parameters failed to show predictive value.

These findings suggest that metabolic disturbances, particularly insulin resistance and hyperuricemia, may contribute to the development of abnormal circadian blood pressure patterns, even in the absence of sustained hypertension. Clinicians should be aware that patients with white-coat hypertension exhibiting these metabolic abnormalities may be at increased risk for non-dipper status and its associated cardiovascular consequences. The presence of elevated uric acid (>5.85 mg/dL in women, >6.15 mg/dL in men) or TyG index (>8.6) in white-coat hypertension patients may serve as warning signs for the non-dipper pattern and warrant closer monitoring of circadian blood pressure patterns.

The evaluation of circadian patterns through morning surge and night–day ratio measurements provides essential data for determining cardiovascular risk levels. The implementation of basic accessible biomarkers in standard practice will help doctors identify at-risk patients. Future prospective studies should investigate whether intervention on these metabolic factors can modify the circadian blood pressure profile.

## Figures and Tables

**Table 1:** Demographic, clinical, laboratory characteristics, and circadian variation in ambulatory blood pressure parameters.

Parameter	All Patients (n = 244)	Dipper (n = 86)	Non-Dipper (n = 158)	*p*-Value
**Demographic and Clinical Features**				
Age (Years) ^a^	32.2 ± 10.1	32.9 ± 10.4	31.9 ± 10.0	0.458
Female, n (%)	177 (72.5)	62 (72.1)	115 (72.8)	0.903
BMI (kg/m^2^) ^a^	25.3 ± 3.1	24.1 ± 2.8	26.0 ± 3.1	<0.001
Smoking, n (%)	84 (34.4)	28 (32.6)	56 (35.4)	0.658
**Ambulatory Blood Pressure Parameters**				
Systolic BP (mmHg)				
Daytime (07:00–23:00) ^a^	–	118.2 ± 8.6	113.2 ± 8.9	<0.001
Nighttime (23:00–07:00) ^a^	–	106.9 ± 8.0	110.4 ± 8.3	<0.01
Nocturnal Fall (%)/Fall ≥ 10% ^a^	–	9.5 ± 3.4/86 (100%)	2.4 ± 4.6/0 (0%)	<0.001/<0.001
Diastolic BP (mmHg)				
Daytime (07:00–23:00) ^a^	–	77.5 ± 7.2	71.6 ± 7.3	<0.001
Nighttime (23:00–07:00) ^a^	–	65.4 ± 6.4	67.0 ± 5.8	<0.05
Nocturnal Fall (%)/Fall ≥ 10% ^a^	–	15.6 ± 3.4/86 (100%)	6.1 ± 6.3/38 (24.1%)	<0.001/<0.001
Mean Arterial Pressure (mmHg)				
Daytime (07:00–23:00) ^a^	–	91.4 ± 7.5	85.5 ± 7.0	<0.001
Nighttime (23:00–07:00) ^a^	–	79.7 ± 7.0	82.4 ± 6.6	<0.01
Nocturnal Fall (%) ^a^	–	12.8 ± 3.2	3.6 ± 5.1	<0.001
Circadian Pattern Characteristics				
Morning Surge—Systolic/Diastolic (mmHg) ^a^	–	11.3 ± 4.2/12.1 ± 3.8	2.8 ± 3.1/4.6 ± 3.4	<0.001/<0.001
Night/Day Ratio—Systolic/Diastolic	–	0.90 ± 0.03/0.84 ± 0.03	0.98 ± 0.05/0.94 ± 0.06	<0.001/<0.001
**Hematologic Parameters**				
Hemoglobin (g/dL) ^a^	14.0 ± 1.4	14.1 ± 1.3	13.9 ± 1.4	0.285
Neutrophil (10^3^/μL) ^a^	3.8 ± 1.2	3.7 ± 1.2	3.9 ± 1.2	0.247
Lymphocyte (10^3^/μL) ^a^	2.2 ± 0.6	2.2 ± 0.6	2.2 ± 0.6	0.892
Platelet (10^3^/μL) ^a^	258 ± 54	259 ± 53	257 ± 55	0.791
**Lipid Profile**				
Total Cholesterol (mg/dL) ^a^	182.3 ± 30.6	175.8 ± 28.7	185.8 ± 31.0	<0.05
HDL-Cholesterol (mg/dL) ^a^	51.3 ± 11.0	53.8 ± 11.4	49.9 ± 10.6	<0.01
LDL-Cholesterol (mg/dL) ^a^	109.4 ± 26.8	103.2 ± 24.5	112.7 ± 27.5	<0.01
Triglyceride (mg/dL) ^b^	108.5 (78–152)	87.5 (65–120)	120.5 (88–165)	<0.001 ^c^
**Biochemical Parameters**				
Fasting Glucose (mg/dL) ^a^	92.8 ± 9.8	91.2 ± 9.1	93.6 ± 10.1	0.067
Creatinine (mg/dL) ^a^	0.85 ± 0.15	0.86 ± 0.14	0.84 ± 0.16	0.352
Uric Acid (mg/dL) ^a^	6.15 ± 0.87	5.65 ± 0.71	6.42 ± 0.83	<0.001
Albumin (g/dL) ^a^	4.2 ± 0.4	4.3 ± 0.4	4.2 ± 0.4	0.128

**Notes:** ^a^ Mean ± standard deviation; ^b^ median (Q1–Q3); ^c^ Mann–Whitney U test; other *p*-values calculated using Student’s *t*-test; normality assessed with Shapiro–Wilk test. Dipper/non-dipper classification was based solely on systolic BP criteria according to the 2018 ESC/ESH guidelines; therefore, some patients classified as non-dippers (due to <10% systolic BP fall) may still exhibit ≥10% diastolic BP fall. ABPM parameters are not presented for “All Patients” column as pooled values across groups with different classification criteria would not be clinically meaningful. **Abbreviations:** BMI, body mass index; BP, blood pressure; HDL, high-density lipoprotein; LDL, low-density lipoprotein.

**Table 2:** Univariate and multivariate logistic regression analyses for the non-dipper pattern.

Variable	Univariate OR (95% CI)	*p*-Value	Model 1 aOR (95% CI)	Model 2 aOR (95% CI)	Model 3 aOR (95% CI)	Model 4 aOR (95% CI)
Age (per year)	0.978 (0.952–1.004)	0.102	0.972 (0.945–1.000) *	–	–	–
Gender (female)	1.052 (0.587–1.885)	0.865	1.038 (0.566–1.903)	–	–	–
BMI (per unit)	1.235 (1.121–1.361) ***	<0.001	1.247 (1.128–1.379) ***	1.186 (1.065–1.321) **	1.183 (1.061–1.319) **	1.158 (1.034–1.297) *
Smoking	1.134 (0.651–1.977)	0.658	–	–	–	–
TyG Index (per unit)	3.842 (2.156–6.847) ***	<0.001	–	2.968 (1.542–5.712) **	2.912 (1.498–5.659) **	2.485 (1.248–4.949) *
AIP (per 0.1 unit)	1.289 (1.126–1.476) ***	<0.001	–	1.892 (0.876–4.087)	–	–
NLR (per unit)	1.089 (0.842–1.408)	0.517	–	–	1.056 (0.798–1.397)	–
PLR (per 10 units)	0.982 (0.925–1.042)	0.548	–	–	–	–
MLR (per 0.1 unit)	1.124 (0.876–1.442)	0.359	–	–	–	–
Uric Acid (per mg/dL)	2.456 (1.712–3.523) ***	<0.001	–	–	–	1.892 (1.267–2.825) **
Nagelkerke R^2^	–	–	0.089	0.198	0.201	0.256

**Notes:** OR, odds ratio; aOR, adjusted odds ratio; CI, confidence interval; BMI, body mass index; TyG, triglyceride–glucose index; AIP, atherogenic index of plasma; NLR, neutrophil-to-lymphocyte ratio; PLR, platelet-to-lymphocyte ratio; MLR, monocyte-to-lymphocyte ratio. * *p* < 0.05, ** *p* < 0.01, *** *p* < 0.001. Candidate variables entered into the stepwise procedure included: age, sex, BMI, smoking status, TyG index, AIP, NLR, PLR, MLR, and uric acid. Variables with *p* < 0.20 in univariate analysis were included in the multivariate model; stepwise entry criterion *p* < 0.05 and removal criterion *p* > 0.10 were applied. Given the high correlation between TyG and AIP (r = 0.909, indicating multicollinearity), AIP was removed through the stepwise procedure due to non-significance when included simultaneously with TyG. **Model definitions:** Model 1: Demographic factors; Model 2: Model 1 + metabolic indices; Model 3: Model 2 + inflammatory indices; Model 4: Model 3 + uric acid. Hosmer–Lemeshow test *p* > 0.05 indicated adequate fit for all models. Final model (Model 4) VIF values: age = 1.12, sex = 1.08, BMI = 1.24, TyG = 1.31, uric acid = 1.19; all values < 3 confirm absence of multicollinearity.

**Table 3:** Subgroup analyses by sex, ROC results, and correlations with nocturnal blood pressure decline.

Parameter	Female (n = 177)	Male (n = 67)	*p*-Value
**Dipper/Non-Dipper Distribution**			
Dipper, n (%)	62 (35.0%)	24 (35.8%)	0.896 ^a^
Non-Dipper, n (%)	115 (65.0%)	43 (64.2%)	
**ROC Analysis for Non-Dipper Prediction**			
Uric Acid			
Cut-off	>5.85 mg/dL	>6.15 mg/dL	
AUC (95% CI)	0.708 (0.632–0.784)	0.745 (0.625–0.865)	
Sensitivity/Specificity	70.4%/66.1%	74.4%/70.8%	
TyG Index			
Cut-off	>8.64	>8.58	
AUC (95% CI)	0.672 (0.593–0.751)	0.712 (0.587–0.837)	
Sensitivity/Specificity	66.1%/64.5%	69.8%/66.7%	
AIP			
Cut-off	>0.43	>0.40	
AUC (95% CI)	0.638 (0.557–0.719)	0.685 (0.556–0.814)	
Sensitivity/Specificity	62.6%/61.3%	67.4%/62.5%	
AUC Comparisons (DeLong Test)			
UA vs. TyG	*p* = 0.031	*p* = 0.045	
UA vs. AIP	*p* = 0.008	*p* = 0.012	
TyG vs. AIP	*p* = 0.142	*p* = 0.176	
**Correlations with Nocturnal BP Decline (Entire Cohort, n = 244)**			
Biomarker	Correlation coefficient (r)		*p*-value
Uric Acid	−0.264		<0.001
TyG Index	−0.177		<0.01
MLR	−0.169		<0.01
AIP	−0.164		<0.05
NLR	−0.086		0.181
PLR	0.046		0.478

**Notes:** AUC, area under the curve; CI, confidence interval; r, Pearson correlation coefficient; UA, uric acid; TyG, triglyceride–glucose index; AIP, atherogenic index of plasma; NLR, neutrophil-to-lymphocyte ratio; PLR, platelet-to-lymphocyte ratio; MLR, monocyte-to-lymphocyte ratio; BP, blood pressure. ^a^ Chi-square test. Cut-off values were determined by the Youden index. AUC differences were tested using the DeLong test with Bonferroni correction for multiple comparisons. Nocturnal BP fall (%) = [(SBP_Day − SBP_Night)/SBP_Day] × 100. Negative correlation indicates higher biomarker values associated with reduced nocturnal BP dipping. Total cohort: dipper 86 (35.2%), non-dipper 158 (64.8%).

**Table 4:** Morning–evening blood pressure comparison and circadian variation in the entire cohort and dipper/non-dipper groups.

Parameter	All Patients (n = 244)	Dipper (n = 86)	Non-Dipper (n = 158)	Within-Group *p*
Systolic BP (mmHg)				
Morning (Mean ± SD)	115.1 ± 9.1	118.2 ± 8.6	113.2 ± 8.9	
Evening (Mean ± SD)	109.2 ± 8.4	106.9 ± 8.0	110.4 ± 8.3	
Morning–Evening Change	Median: 115 → 109 (IQR: 109–121 → 103–115)	Fall: 9.5 ± 3.4%	Fall: 2.4 ± 4.6%	<0.001 */<0.001/0.003
Fall ≥ 10%, n (%)	–	86 (100%)	0 (0%)	
Diastolic BP (mmHg)				
Morning (Mean ± SD)	73.6 ± 7.7	77.5 ± 7.2	71.6 ± 7.3	
Evening (Mean ± SD)	66.5 ± 6.1	65.4 ± 6.4	67.0 ± 5.8	
Morning–Evening Change	Median: 73 → 66 (IQR: 68–79 → 62–71)	Fall: 15.6 ± 3.4%	Fall: 6.1 ± 6.3%	<0.001 */<0.001/<0.001
Fall ≥ 10%, n (%)	–	86 (100%)	38 (24.1%)	
Mean Arterial Pressure (mmHg)				
Morning (Mean ± SD)	87.5 ± 7.7	91.4 ± 7.5	85.5 ± 7.0	
Evening (Mean ± SD)	81.5 ± 6.9	79.7 ± 7.0	82.4 ± 6.6	
Morning–Evening Change	Median: 87 → 81 (IQR: 82–93 → 76–86)	Fall: 12.8 ± 3.2%	Fall: 3.6 ± 5.1%	<0.001 */<0.001/<0.001
Pulse Pressure (mmHg)	41.5 ± 8.2 → 42.7 ± 7.8	–	–	0.087
Categorical Variables (Morning/Evening)				
SBP ≥ 140 mmHg, n (%)	8 (3.3%)/2 (0.8%)	–	–	0.039 †
DBP ≥ 90 mmHg, n (%)	11 (4.5%)/3 (1.2%)	–	–	0.021 †
Between-Group Comparisons (Dipper vs. Non-Dipper)		Morning *p*	Evening *p*	Fall % *p*
Systolic BP		<0.001	<0.01	<0.001
Diastolic BP		<0.001	0.042	<0.001
Mean Arterial Pressure		<0.001	<0.01	–

**Notes:** SD, standard deviation; IQR, interquartile range (Q1–Q3); SBP, systolic blood pressure; DBP, diastolic blood pressure; BP, blood pressure. Data are presented as mean ± SD for normally distributed variables and median (IQR) for non-normally distributed variables. Within-group *p*-values shown as: all patients/dipper/non-dipper. * Paired *t*-test or Wilcoxon signed-rank test; † McNemar test. Between-group comparisons were made using independent *t*-tests. Statistical significance was set at *p* < 0.05.

## Data Availability

The data presented in this study are available on request from the corresponding author. The data are not publicly accessible due to privacy restrictions.

## References

[1] Manea V., Leucuţa D.C., Pop C., Popescu M.I. (2024). The biomarkers associated with non-dipper pattern in patients with type 2 diabetes with hypertension. Med. Pharm. Rep..

[2] Karayiğit O., Dolu A.K., Çelik M.C., Özkan C., Demirtaş B. (2023). Relationship between the Atherogenic Index of Plasma and Nondipping Circadian Pattern in Hypertensive Patients. Med. Princ. Pract..

[3] Hermida R.C., Ayala D.E., Mojón A., Fernández J.R. (2013). Blunted sleep-time relative blood pressure decline increases cardiovascular risk independent of blood pressure level–the “normotensive non-dipper” paradox. Chronobiol. Int..

[4] Du Y., Zhu B., Liu Y., Zhou W., Du Z., Yang W., Gao C. (2024). Association between nocturnal blood pressure phenotype and adverse cardiovascular prognosis in patients with coronary heart disease and hypertension. J. Clin. Hypertens..

[5] Nkoke C., Jingi A.M., Noubiap J.J., Teuwafeu D., Nkouonlack C., Gobina R., Djibrilla S., Abas A., Dzudie A. (2022). Gender Differences in Cardiovascular Risk Factors, Clinical Presentation, and Outcome of Patients Admitted with a Hypertensive Crisis at the Buea Regional Hospital, Cameroon. Int. J. Hypertens..

[6] Maas A.H., Appelman Y.E. (2010). Gender differences in coronary heart disease. Neth. Heart J..

[7] Mattioli A.V., Coppi F., Nasi M., Pinti M., Gallina S. (2022). Long COVID: A New Challenge for Prevention of Obesity in Women. Am. J. Lifestyle Med..

[8] Najman J.M., Kisely S., Scott J.G., Ushula T.W., Williams G.M., Clavarino A.M., McGee T.R., Mamun A.A., Wang W.Y.S. (2024). Gender differences in cardiovascular disease risk: Adolescence to young adulthood. Nutr. Metab. Cardiovasc. Dis..

[9] Lee E.M. (2024). When and how to use ambulatory blood pressure monitoring and home blood pressure monitoring for managing hypertension. Clin. Hypertens..

[10] Faraci F.M., Scheer F.A.J.L. (2024). Hypertension: Causes and Consequences of Circadian Rhythms in Blood Pressure. Circ. Res..

[11] Şaylık F., Çınar T., Selçuk M., Akbulut T. (2022). Triglyceride-to-glucose index to detect a non-dipping circadian pattern in newly diagnosed hypertensive patients. J. Cardiovasc. Thorac. Res..

[12] Özyaşar M., Memioğlu T. (2025). Comparative analysis of systemic inflammatory biomarkers on dipper and non-dipper hypertension phenotypes. Medicine.

[13] Williams B., Mancia G., Spiering W., Rosei E.A., Azizi M., Burnier M., Clement D.L., Coca A., de Simone G., Dominiczak A. (2018). 2018 ESC/ESH Guidelines for the management of arterial hypertension. Eur Heart J..

[14] McEvoy J.W., McCarthy C.P., Bruno R.M., Brouwers S., Canavan M.D., Ceconi C., Christodorescu R.M., Daskalopoulou S.S., Ferro C.J., Gerdts E. (2024). 2024 ESC Guidelines for the management of elevated blood pressure and hypertension. Eur. Heart J..

[15] Sun T., Liu J. (2025). Study on the correlation between triglyceride glucose index, triglyceride glucose index to high-density lipoprotein cholesterol ratio, and the risk of diabetes in nonalcoholic fatty liver disease. Front. Endocrinol..

[16] Asil S., Barış V.Ö., Eravcı Ö., Taşkan H., Çelik M., Yüksel U.Ç. (2022). Relationship Between Ambulatory Blood Pressure Variability and Atherogenic Index of Plasma. Eur. J. Cardiovasc. Med..

[17] Durak M.İ. (2024). The Relationship Between Ambulatory Blood Pressure Monitoring and Uric Acid Level in Hypertensive Patients. Celal Bayar Üniversitesi Sağlık Bilim. Enstitüsü Derg..

[18] Zhu J., Shen L., Jia S., Wang W., Xiong Y. (2024). The role of uric acid in the risk of hypertension developed from prehypertension: A five-year Chinese urban cohort study. Arch. Public Health.

[19] Lv Y., Cao X., Yu K., Pu J., Tang Z., Wei N., Wang J., Liu F., Li S. (2024). Gender differences in all-cause and cardiovascular mortality among US adults: From NHANES 2005–2018. Front Cardiovasc. Med..

[20] Chotruangnapa C., Tansakun T., Roubsanthisuk W. (2021). Clinical risk factors and predictive score for the non-dipper profile in hypertensive patients: A case-control study. Clin. Hypertens..

[21] Huart J., Persu A., Lengelé J.-P., Krzesinski J.-M., Jouret F., Stergiou G.S. (2023). Pathophysiology of the nondipping blood pressure pattern. Hypertension.

[22] Chu Y.H., Sun Z.J., Chang Y.F., Yang Y.-C., Chang C.-J., Chou Y.-T., Wu J.-S. (2023). Different factors associated with morning blood pressure surge in antihypertensive-naïve dipper and non-dipper subjects. J. Clin. Med..

[23] Renna N.F., Ramirez J.M., Murua M., Bernasconi P.A., Repetto J.M., Verdugo R.A., Farez B.G., Miatello R.M., Diez E.R. (2023). Morning blood pressure surge as a predictor of cardiovascular events in patients with hypertension. Blood Press. Monit..

[24] Havelkova A., Dvorak P., Siegelova J., Dobsak P., Filipensky P., Cornelissen G. (2023). Possibilities of interpreting the night-to-day ratio specified by 24-hour blood pressure monitoring. Int. J. Clin. Pract..

[25] Perticone M., Maio R., Shehaj E., Gigliotti S., Caroleo B., Suraci E., Sciacqua A., Andreozzi F., Perticone F. (2023). Sex-related differences for uric acid in the prediction of cardiovascular events in essential hypertension: A population prospective study. Cardiovasc. Diabetol..

[26] Liu D., Zheng X., Zhu J., Yang J., Lu L., Ji X., Hui J., Luo Y. (2025). Gender-specific association between serum uric acid levels and hypertension in East China: A cross-sectional study. BMC Public Health.

[27] Kuwabara M., Niwa K., Hisatome I., Nakagawa T., Roncal-Jimenez C.A., Andres-Hernando A., Bjornstad P., Jensen T., Sato Y., Milagres T. (2017). Asymptomatic Hyperuricemia Without Comorbidities Predicts Cardiometabolic Diseases: Five-Year Japanese Cohort Study. Hypertension.

